# Physical demands of collegiate basketball practice: a preliminary report on novel methods and metrics

**DOI:** 10.3389/fspor.2024.1324650

**Published:** 2024-09-23

**Authors:** Peter Huynh, Samuel Guadagnino, Jessica Zendler, Cristine Agresta

**Affiliations:** ^1^ Rehabilitation Medicine Department, University of Washington, Seattle, WA, United States; ^2^ Rimkus, Houston, TX, United States; ^3^ School of Kinesiology, University of Michigan, Ann Arbor, MI, United States

**Keywords:** return-to-sport, time-motion analysis, loading, position, movement

## Abstract

Knowing the specific physical demands of basketball players can provide useful information for clinical decision making when rehabilitating athletes following injury. The purpose of this observational study was to describe the physical demands of basketball play at the Division I collegiate level using video-based time-motion analysis and introduce a time-efficient alternative method of quantifying demands. Eleven NCAA Division I basketball players (6M, 5F; 4 guards, 4 centers, 3 forwards) participated in the study. Video footage was collected from four practices (2 men's, 2 women's) and used to quantify the types and frequencies of player movements based on definitions from seminal work. A second and simpler method was also used to classify movement. A two-way ANOVA was used to assess significant differences in movement by team (men's, women's) and position (guard, forward, center). There were significant differences in counts of stand/walk (*p* < 0.001), jog (*p* = 0.012), run (*p* = 0.001), stride/sprint (*p* = 0.04), and medium-intensity shuffling (*p* < 0.001) per minute and proportion of practice time spent in bodyweight (*p* < 0.001) or above-bodyweight (*p* < 0.001) loading between teams. There were significant differences for jog (*p* = 0.001) and transition (*p* = 0.07) rates across positions. Position and team are important considerations for rehabilitation and return-to-sport clearance. Quantification of these demands can be reliably acquired through video analysis using a simplified method (estimated foot load) or using traditional methods of movement classification and counts, particularly when applying descriptors that better capture the current style of play.

## 1 Introduction

At the high school level, approximately 430,000 girls and 550,000 boys play interscholastic basketball (National Federation of State High School Association) with a small percentage advancing to the collegiate level. Injuries are highly prevalent in basketball at all competitive levels (1). Position (2) and gender (3) seem to influence the type and frequency of injury. For example, while lower injuries are most prominent for both genders, male collegiate players had a higher rate of sprain injuries in practice while women collegiate had a higher rate of overuse injuries in practice (3). Considering competition and practice scenarios, ankle sprains were most common in both genders, but more occurred more frequently in male players (3). Guards have a higher percentage of knee injuries compared to forwards or centers (4, 5). Differences in the physical demands across positions and styles of play between gender (6–9) may contribute to differences in injuries. Thus, quantification of movements to classify physical demands and determine style of play (i.e., speed, frequency of movement) has become popular for helping to craft training programs to ensure appropriate fitness for play and to reduce risk of injury (10). In addition to training, an improved understanding of physical demands by position and gender could help to guide rehabilitation and return-to-sport (RTS) decision making by tailoring treatment to positional and/or team normative values. Such information would clarify the nature, intensity, and frequency of movements required for their specific skill level, position, and gender. To date, there are few data-supported recommendations to increase precision around RTS decisions, even for highly prevalent injuries like lateral ankle sprains (11, 12). The impact of this lack of clinical guidance is evident by the high-reinjury rates among players (5, 13).

Athlete tracking systems that use sensors to classify movements and quantify movement intensity have exploded at the elite levels of sport (14). These technologies typically employ global navigation satellite system (GNSS), local positioning system (LPS), and/or inertial measurement unit (IMU) technology to collect whole-body movement data, such as speed, distance, and acceleration, from a single sensor. They also quantify proprietary measures of “player load” and track events such as jumps and changes of direction, although with varying and proprietary definitions. While using athlete tracking technology to quantify “player load” greatly reduces the labor cost associated with traditional classification methods, like video time-motion analysis, there is still a need to use (and improve) video-based time-motion analysis, because (1) non-elite programs, like high school sport teams and lower division collegiate programs, may not have the financial or personnel resources to effectively incorporate tracking systems to monitor their athletes and (2) the discrete events and movements defined by video analysis represent unique data from those determined by athlete tracking technologies, with the former being defined by visualization of a whole movement and the latter being an estimate based on velocity and/or acceleration at a single point on the athlete's body. Furthermore, the definitions of movements and events used by athlete tracking technologies vary widely depending on the technology manufacturer and are largely unvalidated with respect to ground truth. This is particularly true with respect to rehabilitation management, where knowledge of discrete movements and frequencies is more intuitive for clinicians to understand and utilize compared to signals or outputs from sensor technology. Considering that prior injury is a consistent predictor of subsequent injury (15), improving rehabilitation strategies to prevent primary injuries or reduce secondary injuries could provide long-term benefit to many players.

The purpose of this brief research report was to categorize the physical demands of in-season practices for a women's and a men's NCAA Division I basketball team using video-based time-motion analysis. Because manually classifying movements from video footage is a major barrier to effectively using video time-motion analysis for real-time rehabilitation decisions, we secondarily introduced a novel and more efficient method of analyzing video data to estimate physical demands as an alternative to traditional video time-motion analysis. As proof-of-concept we provide inter-rater reliability values for the novel vs. traditional methods as well as preliminary descriptive values (in supplemental content) for the physical demands, including movement transitions, of select collegiate basketball players.

## 2 Materials and methods

### 2.1 Participants

The study was a preliminary proof-of-concept study for assessing movement demands using a traditional and a novel, simplified method. A convenience sample of 11 collegiate players (6M, 5F; 4 guards, 4 centers, 3 forwards) competing in NCAA Division I basketball were recruited to participate in this study. The players were all members of the same university's men's and women's basketball teams, respectively. The University Institutional Review Board approved all procedures prior to data collection. Players provided informed consent to participate in the study.

### 2.2 Video recordings

Video footage was collected from four practices in total: two for the men's team and two for the women's team. Each practice was similar in character and recorded at similar times of the week and season. Practice 1 for the men's and women's teams was recorded two days before a regular-season game and incorporated a separate component of running or sprinting as player conditioning. Practice 2 for the men's and women's teams was recorded the day before a regular-season game. It was shorter in duration and did not include any running or sprints separate from scrimmage drills, likely to allow for appropriate player rest prior to competition. Activities in practice occurred in half-court and full-court scenarios and included dynamic warm-ups, shooting, agility drills, 5v5 drills, walk-throughs, sprints, and short film sessions (Supplementary Table S1). All practices were recorded during the middle of the regular season.

We employed two video cameras (GoPro Hero9 SPBL1, GoPro San Mateo, CA, USA; 30 Hz) to capture footage of each practice, based on previous methods (7, 8, 16–23). One camera was placed at half-court along the sideline and the other at the baseline. The cameras were placed at a vantage point where all players on the court were visible in the same view. Photos of each player were taken with their jersey number and last name and were referenced during video coding to allow for ease of locating during video analysis. Video footage from both cameras were manually synchronized during analysis by identifying the same event in both cameras and recording the offset between time stamps for the start of the event.

#### 2.2.1 Modified McInnes movement classification including transitions

Video was reviewed using Windows Media Player (Microsoft Corporation, Redmond, WA, USA) or QuickTime (Apple Inc., Cupertino, CA, USA) to quantify the types and frequencies of player movements based on well-accepted movement definitions (10). Three raters examined footage frame-by-frame as needed and recorded the frequencies and time stamps of each movement observed. Raters manually coded movements using the following eight categories: (1) standing/walking, (2) jogging, (3) running, (4) striding/sprinting, (5) low-intensity shuffling, (6) medium-intensity shuffling, (7) high-intensity shuffling, and (8) jumping. In pilot testing, we found some ambiguity in distinguishing categories when using the original (10) definitions. Therefore, we incorporated additional descriptors to the definitions to improve specificity by better capturing current basketball style of play. Since transitioning between movements typically involves acceleration and/or change of direction and may place an additional loading demand on the body, we explicitly recorded *transitions* and added transitions as a movement classification. Transitions were defined as movements occurring briefly between one designated movement category to another (e.g., pivoting, cutting, and quick side shuffles of no more than two steps). See Table 1 for a complete list of movement classifications for this method (“modified McInnes”) with operational descriptions.

**Table 1:** List of eight movement types coded from video and operational descriptors used to determine movement type.

Type	Operational descriptors^a^
Stand/walk	- Activity**/steps** at no greater intensity than walking. No distinction between standing and walking. - No distinction made between standing and walking or between different intensities of walking. - Includes instances when a player is in a defensive stance: **either stationary or taking steps at an intensity no greater than walking.** - **Steps can be taken in any direction: forward, backward, or lateral but no shuffling occurs.** - **No flight phase (both feet never leave the ground).** - **Includes an offensive player performing a pick or a defensive player taking a charge. Both feet are planted in these cases.** - **Includes instances where a player is standing or stepping (but not shuffling) with no flight phase to hold/gain position underneath the net.**
Jog	- Movement (forward or backward) at intensity greater than walking but without urgency - **Includes skipping** - **Includes cross-over running, movement laterally with feet-crossing over each other, without urgency** - **Often moving in a general direction rather than a clearly defined target** - **Sharp change in direction can be achieved at this speed without deceleration or a lateral cutting movement** - **Upright torso angle**
Run	- Forwards/backwards movement at intensity greater than jog and a moderate degree of urgency but not approaching intense level of movement - **Includes cross-over running, movement laterally with feet-crossing over each other, with urgency** - **Often marked by moving toward a clearly defined target** - **Sharp change in direction cannot be achieved at this speed without deceleration or a lateral cutting movement** - **Torso can be either upright or slightly leaned forward**
Stride/sprint	- Forward movement at high intensity, effort and purpose at or close to maximum - **Increased knee drive near or past 90 degrees hip flexion** - **Often marked by dramatically increased forward trunk angle**
Low intensity shuffle	- Lateral or backward movement using shuffling action of feet - Without urgency, slow rate of foot movement and erect posture - **Often moving in a general direction rather than a clearly defined target**
Medium intensity Shuffle	- Shuffling at medium intensity with moderate level of urgency - Moderate rate of foot movement but not approaching an intense level of shuffling-type movement - **Often marked by moving with or toward a clearly defined target** - **Posture can be erect or slight squat position** - **Speed does not exceed the player’s jogging speed**
High intensity shuffle	- Shuffling at high intensity characterized by effort/urgency and rapid foot movement while usually in squat position - **Speed and urgency approach a range near, at, or past the player’s running speed. Player should be able to seamlessly transition into running (e.g., faster than the player’s jogging speed).** - **Includes instances where a player is driving into the other player while shuffling their feet** - Ground may not have been covered as the feet may have been shuffling rapidly on the spot or transferring weight from side to side
Jump	- Time from initiation of jump to completion of landing - **Both feet leave the ground with the player showing intent to increase their playing height and/or move vertically**

Bold text indicates modifications and/or additions made to descriptors from original work of McInnes et al. (10) to improve classification and level of agreement among raters.

^a^ We updated the McInnes definitions to provide specific descriptors that could allow raters to better discriminate varying intensities of movement patterns. For example, the differences between running and striding/sprinting were originally defined with descriptors such as “moderate degree of urgency” and “high intensity”. However, the subjective nature of these descriptors applied in varying contexts left situations open to varied interpretation between raters. Adding specific descriptors for trunk lean, hip flexion angle, and movement toward clearly defined targets vs. a general direction allowed raters to report their findings with a higher degree of reliability. Reference: McInnes SE, Carlson JS, Jones CJ, McKenna MJ. The physiological load imposed on basketball players. *Journal of Sports Sciences*. 1995;13:387–397.

#### 2.2.2 Novel foot loading movement classification

The video-based time-motion analysis method, even with the modified definitions, was highly time intensive. Therefore, we developed a second and simpler movement classification system based on estimated magnitude of load at the feet. This novel movement classification (“foot loading”) provided a simpler, more efficient, and more readily accessible method of analyzing movements compared to traditional methods and provided clinically meaningful information for considering RTS following (lower body) injury. Player actions were classified into one of three foot-loading categories: below bodyweight (B-BW), bodyweight (BW), and above bodyweight (A-BW). B-BW was defined as any activity that unweighted the load of a player's body off their feet (e.g., sitting, lying, kneeling). BW was defined by standing/walking activities, per previous work (10) because these activities generally produce ground reaction force magnitudes approximately equal to bodyweight (24). A-BW was defined as any movement more intense than standing/walking (e.g., jogging, running, jumping) (Table 2).

**Table 2:** Simplified categories of movement in relation to generalized body load and operational definitions used to visually assign movements into categories.

Movement category	Operational descriptors
Below bodyweight (B-BW)	- Activities that unweight the usual load of a player's body off their feet, which includes sitting, horizontal/lying on the ground (e.g., following a fall to the ground), or kneeling
Bodyweight (BW)	- Activity at no greater intensity than walking. Steps can be any direction: forward, backward, lateral. If steps are lateral, feet do not shuffle. See shuffle definition below for reference. - Includes leaning on an object (e.g., a stanchion or table) - Includes instances when player is in a defensive stance but not moving. - No flight phase: both feet never leave the ground. - Any time the player is off view from the camera or not participating in the drill and view is obstructed by the stanchion
Above bodyweight (A-WB)	- Any movement not within the definition of standing/walking or bench/sitting, which includes, pivoting, transitional movements/changing direction (e.g., cutting, jump-cuts), driving into another player, jogging, skipping, running, sprinting, shuffling (i.e., one foot replaces the position of the other within rough proximity), and jumping

Movement categories are based on estimated magnitude of ground reaction force loading at the feet in relation to bodyweight.

### 2.3 Statistical analysis

Counts of movements classified per the modified McInnes categories were calculated for each player and each practice. However, since the foot loading categories were fewer and more broadly defined, it seemed more appropriate to quantify duration spent in each category by player and practice. Movement-category frequency counts and durations were normalized to practice time in minutes for comparison across practices and teams. To assess interrater reliability, all three raters independently coded six players for the same 700 s video clip. Intraclass correlation coefficients of the movement counts and durations were assessed for both movement classifications schemes. For context, a two-way ANOVA was used to assess differences in movements across position and team. Pairwise comparisons were examined via Tukey's HSD test when significant main effects were found without the presence of a significant interaction. A Shapiro-Wilk Test was performed by gender for each outcome of interest to confirm data normality. A Levene Test was performed for each outcome of interested to confirm homogeneity of variance. Significance was set as *a priori* at alpha <0.05.

## 3 Results

### 3.1 Inter-rater reliability

Results of the inter-rater reliability assessment can be found in supplemental files (supplemental content, Supplementary Table S2). Overall, all categories had excellent reliability (ICC > 0.70) except runs (0.63) and high intensity shuffles (0.43).

### 3.2 Practice exposure

Total practice exposure across the two recorded sessions differed between the men's (approx.189 min) and women's teams (approx. 227 min). Within both teams, practice 1 was substantially longer than practice 2 (Supplementary Table S1). Thus, all subsequent analyses used time-normalized movement counts (modified McInnes) and durations (foot loading). Within each team, total practice minutes were calculated by summing practice 1 and practice 2 durations. Likewise, the outcome measures were summed across practices for each player and divided by the appropriate total practice minutes. Modified McInnes movement counts were expressed as a movement rate (count per minute) and foot loading durations were expressed as proportion of total practice time. Absolute (Supplementary Tables S3, S4) values and aggregate descriptive values (Supplementary Tables S5, S6) can be found in the supplemental content.

### 3.3 Modified McInnes movement classifications

#### 3.3.1 Team differences

We found a significant main effect of team for rates of stand/walk [F(5,1) = 46.39, *p* < 0.001, d = 2.7], jog [F(5,1) = 8.15, *p* = 0.012, d = −0.85], run [F(5,1) = 26.81, *p* = 0.001, d = 2.4], stride/sprint [F(5,1) = 20.00, *p* = 0.04, d = 2.9], medium intensity shuffling [F(5,1) = 50.29, *p* < 0.001, d = 2.8], high-intensity shuffling [F(5,1) = 6.72, *p* = 0.02, d = 1.6] and transitions [F(5,1) = 31.50, *p* < 0.001, d = 2.4]. The women's team had a lower rate of each of these movement types compared to the men's team, except for jog, in which the women significantly exceeded the men.

#### 3.3.2 Position differences

**Figure 1:**
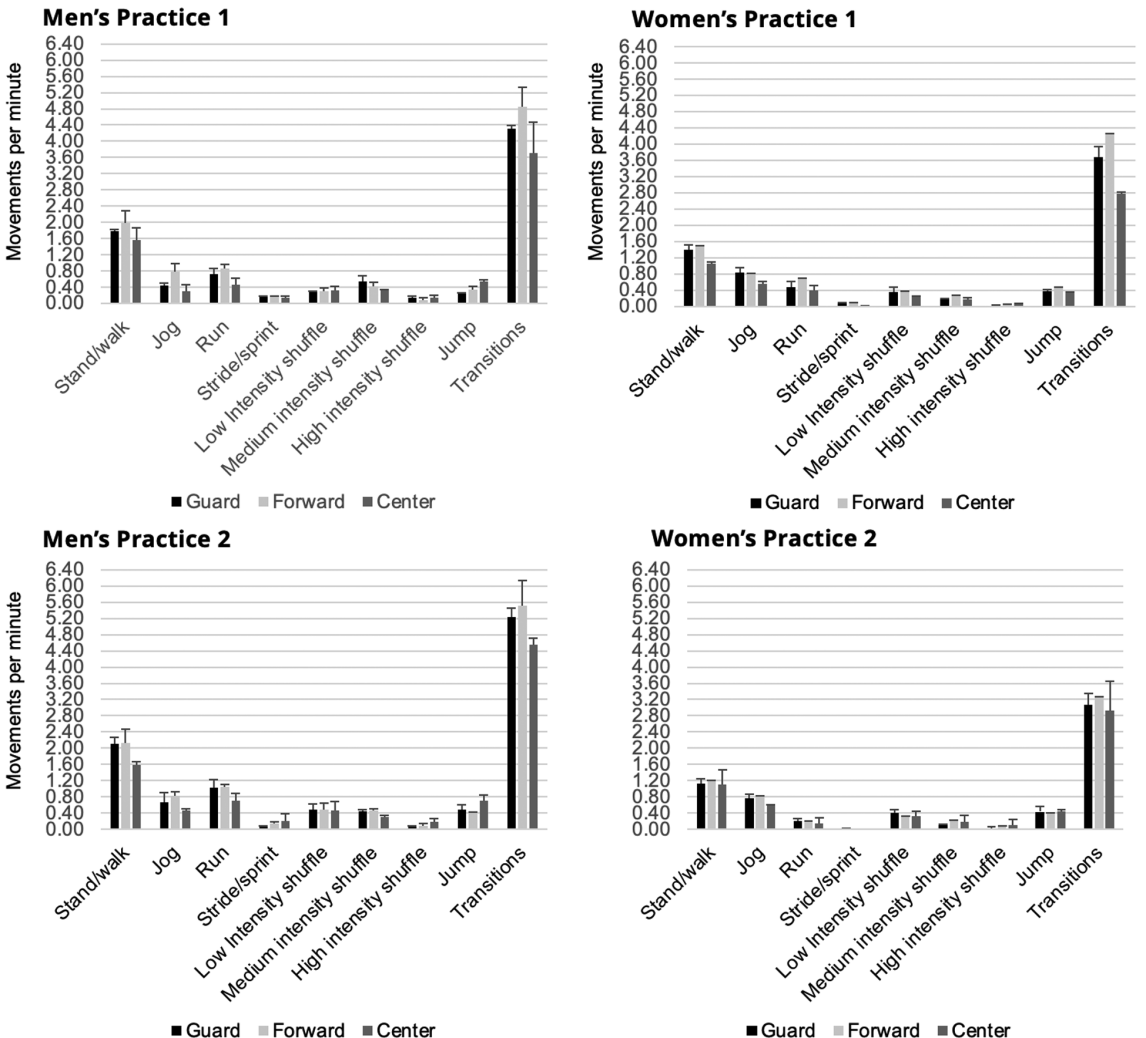
Movement counts normalized to practice duration (movements per minute) by team and position. Thick bars represent the mean across players and practice sessions. Thin bars indicate the one standard deviation from the mean.

We found a significant main effect of position for stand/walk [F(5,2) = 6.03, *p* = 0.011, d = 0.94]. Centers, regardless of team, had lower rates of stand/walk compared to forwards. However, this comparison did not reach statistical significance (*p* = 0.070). We found a significant main effect of position for jog [F(5,2) = 11.95, *p* = 0.007, d = 0.97]. Centers, regardless of team, had lower rates of jogging compared to forwards (*p* = 0.002) or guards (*p* = 0.052). We also found a significant main effect of position for transitions [F(5,2) = 0.031, d = 0.07] with centers having a lower rate of transitions compared to forwards (*p* = 0.047). We found a significant interaction between team and position for medium-intensity shuffling [F(5,2) = 5.19, *p* = 0.028, d = 0.93] and jumps [F(5,2) = 5.05, *p* = 0.02, d = 0.93] (Figure 1). It is important to note that of frequency variables examined, homogeneity of variance was not met for both jump (*p* = 0.028) and medium-intensity shuffling (*p* = 0.046).

### 3.4 Foot loading movement classification

#### 3.4.1 Team differences

We found a significant main effect of team for proportion of practice in B-BW [F(5,1) = 113.82, *p* < 0.001, d = 1.0], BW [F(5,1) = 531.31, *p* < 0.001, d = 1.0] and A-BW [F(5,1) = 570.73, *p* < 0.001, d = 1.0] movements, with the women’s team having lower proportion of time spent in BW and A-BW.

#### 3.4.2 Position differences

**Figure 2:**
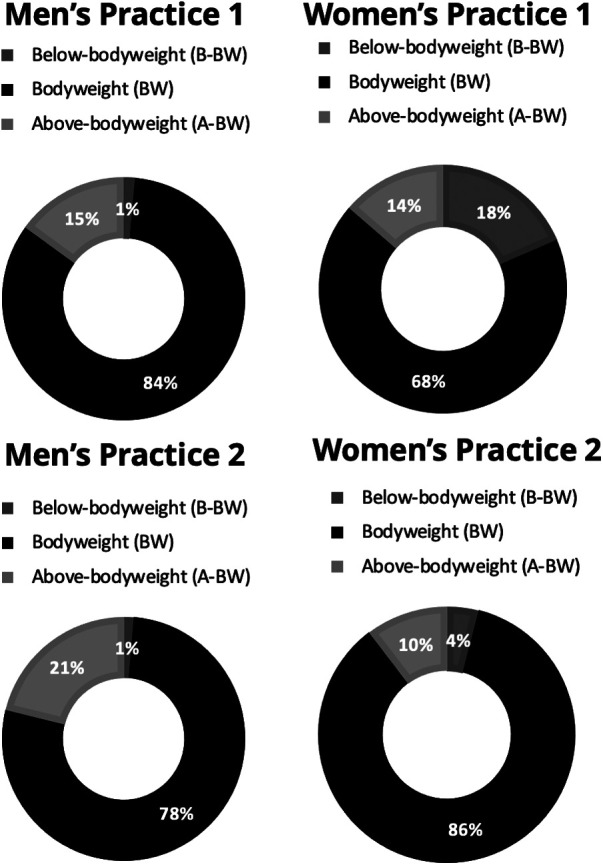
Percentage of practice time spent in movements estimated to produce foot loads below bodyweight (B-BW), around bodyweight (BW), or above bodyweight (A-BW) by position and team.

We found a significant interaction between team and position for proportion of practice in B-BW (Figure 2).

## 4 Discussion

The purpose of this study was to categorize the physical demands of in-season practices for a women's and a men's NCAA Division I basketball team. We used video-based time-motion analysis to evaluate movement frequency and duration by position across two typical practices for each team. We used a previously published method (10), with some modifications to improve specificity, to get movement frequencies across eight categories of basketball movement. We also tabulated the number of transitions between movement types. We developed a simplified classification of movement related to estimated load at the feet to provide a rule-of-thumb approximation of movement demand. We used this to classify time spent in activities producing below (sitting/lying), at (walking, standing), or above bodyweight (anything more vigorous than walking) foot loads. We found significant differences in several outcome measures by team and position, which highlights that rehabilitation strategy and RTS criteria may need to be tailored to the player's unique playing demands based on their team practice style and position.

### 4.1 Comparison between teams

The men's and women's teams differed significantly across most movement categories except for rates of low intensity shuffling and jumps. Men's players performed significantly higher rates of standing/walking, running, striding/sprinting, medium-intensity shuffling, and transitions and significantly lower rates of jogging compared to women's players. Likewise, the men's teams had significantly higher proportion of practice time spent in BW or A-BW loading. These findings differ from similar studies comparing males and females at the junior and NCAA Division II level (8, 17), which found movement patterns to be similar between genders. Differences in results between our study and previous ones may be due to the types of events observed. Specifically, previous studies gathered data from practice games (2 vs. 2, 4 vs. 4, or 5 vs. 5) or competitions whereas our study examined typical in-season practices that incorporated drills in addition to game-like activity. Additionally, differences in practice plans, upcoming competition, practice duration, coaching staff, and individual playing styles in our study may have influenced results.

Results from this study suggest that the men's team had a different practice style than the women's team. The higher frequency of low (stand/walk) and high-intensity movements (run, stride/sprint) for the men's team suggests their practices were characterized by intermittent high intensity bursts and recovery. Low-intensity (stand/walk) activity may have been necessary to rest between bouts of high-intensity activity (run and stride/sprint). Conversely, the women's team spent more time in low to moderate movement intensities as demonstrated by longer proportions of time spent in B-BW loading and significantly higher frequencies of jogging. Considering that female players experience higher rates of overuse injuries during practice (3), monitoring the ratio of B-BW to A-BW time would be important for players returning from repetitive stress injuries. Additionally, the men's practices were shorter in duration than the women's practices, but the men's players performed a significantly higher number of total movements and a greater number of transitions. The pace of men's basketball has been suggested to be faster than women's (6, 10, 16, 20). Our results seem to align with this observation, but since we only examined one team of each gender and only a snapshot of practices, it is impossible to parse out how much of the difference is due to gender, to differing training philosophies, or simply to chance.

### 4.2 Comparison between positions

Some differences were found between positions, although fewer than were found between teams. Within both teams, centers jogged significantly less than forwards. Transitions also depended on position. While pairwise comparisons were not significant, our data indicates that forwards made more transitions than centers or guards. Although findings did not reach statistical significance (*p* = 0.059), centers ran less frequently than forwards or guards. Additionally, significant interactions of team and position were found for jumps and B-BW loading. Men's centers jumped more than other positions whereas women's positions had similar jump rates. Conversely, women's centers had more B-BW compared to other positions while men's players had similar proportions of B-BW.

While our findings used time-normalized movement counts and proportions rather than absolute values, the findings are consistent with similar studies and suggest that positional differences exist at the junior, college, and professional level (6, 9, 18, 20, 23, 25, 26). However, to our knowledge, this is the first study that examines the interaction of position and team (gender) on movement frequencies and durations. The interaction found for jumping rates highlights that importance of analyzing within team and that positional differences should not be assumed across all teams or genders. Furthermore, context should be considered when assessing movement demands. The total number of jumps for women's players during Practice 1 (53.2 ± 9.2) were influenced by an agility drill that required players to jump consecutively seven times through miniature hurdles. Had this drill not been conducted, total frequency of jumps for the women's players may have been similar to jumps for the men's players, possibly eliminating a significant interaction. This observation highlights the fact that the coach's plan for each practice can have a significant effect on player loading across teams or between points of the week or season. Therefore, careful investigation of the athlete's unique playing situation is advisable when devising a rehabilitation or RTS strategy.

### 4.3 Novel movement classification offers an efficient first-pass approximation of RTS loads

A significant contribution of this work is the development of a more efficient and reliable video-based movement classification system. Video movement analysis using the McInnes definitions was a time-intensive process, and we found the inter-rater reliability varied depending on movement type. With RTS in mind and understanding that few clinical practitioners have the time or resources to execute traditional time-motion analysis, we deployed a secondary analysis aimed at rapidly estimating movement demands that we would expect to influence rehabilitation progression or RTS criteria. Namely, we approximated foot loading based on whether the activity would elicit vertical ground reaction forces below, at, or above bodyweight because modulation and progression of weight bearing time and intensity is one of the most common and immediately employed rehabilitation treatments following lower body injury. Further, weight-bearing functional activities would be expected to require more muscle strength, coordination, and balance, and therefore be more taxing on the musculoskeletal system, than the sitting, lying, and kneeling activities associated with the below-bodyweight classification. Our novel approach using estimated foot loading relative to bodyweight (B-BW, BW, and A-BW) offers a first-pass approximation for clinical decision making, particularly around lower-body injury (e.g., returning from foot fracture). For instance, the duration of bodyweight loading in combination with an athlete's physical signs and reported symptoms can determine current healing/functional status and provide objective benchmarks for sport progression or clearance. Given that our preliminary findings with traditional time-motion analysis suggest that the player's unique team and position demands may warrant consideration when devising a RTS strategy, this simplified method would permit a clinician to more effectively progress through rehabilitation or clear a player for practice based on review of inexpensive video footage of a few practices without the significant labor-hours required of traditional analyses. The application of known differences in McInnes-classified movements when assessing bodyweight movement durations can be a powerful tool for clinical decision making. For example, the A-BW time for male centers is likely spent jumping whereas forwards’ time spent is likely transitioning between movements, which involves frequent change of directions. Centers recovering from foot fractures or Achilles tendon injuries may need to limit practice time compared to guards due to the high loads on bone and strain on tendon from jumping. Likewise, monitoring A-BW time for forwards returning from ACL or ankle sprains may help to limit change-of-direction movements during play. This could be particularly advantageous in situations where player tracking devices (e.g., LPS, IMU) are not available for the team and the clinician must make an educated guess on what types of loads the player must sustain. Even in the case of player tracking devices being available, cross-referencing the player tracking data to some exemplar time-motion data using the foot loading method provides greater context for decision-making with respect to player tracking data.

### 4.4 Modified McInnes definitions

The use of modified McInnes definitions improved efficiency of rater classification by reducing ambiguity of movement descriptions. We updated the McInnes definitions to provide specific descriptors that could allow raters to better discriminate varying intensities of movement patterns. We found these definitions to be particularly helpful for coders, such as student assistants, unfamiliar with basketball play. For example, the differences between running and striding/sprinting were originally defined with descriptors such as “moderate degree of urgency” and “high intensity”. However, the subjective nature of these descriptors left situations open to variable interpretation among raters. Adding specific descriptors for trunk lean, hip flexion angle, and movement toward clearly defined targets allowed raters to report their findings with a higher degree of objectivity. When more specific data related to speed and/or direction of movement is required, we recommend using the modified McInnes definitions presented herein.

### 4.5 Limitations

A critical limitation of this study is the small sample size of players and teams. We provided a strong, small-scale comparison of team and position by recruiting men and women from the same university and level of play and by gathering data on the same 11 players from similar times of the week and season. However, these data represent only a snapshot of specific teams and should not be assumed to be representative of all collegiate basketball. Likewise, having only one team of each gender precludes extensive discussion of movement demands by gender. As these data represent practices only, game data would need to be collected to extend decision-making to return to full gameplay.

Several more factors that may influence physical demands which were not explored in this study include playing style, coaching staff, presence of player injuries, timepoint in the season, and geographic location within the United States. Likewise, the small sample size left the study underpowered for further statistical analysis, including analysis by practice session or within practice drills. Additionally, our study relied on raters’ subject assessment of the movement and how best it fit with our qualitative descriptions of each movement type. More rigorous methods of classification (9) may influence differences found between positions and gender. Future studies should continue to uncover the physical demands pattern across micro- and macro-cycles or between healthy players vs. those returning from injury as this knowledge could improve forecasting of injury susceptibility and decisions around which practices injured athletes can attend as they rehabilitate.

### 4.6 Practical application

Our findings illustrate that differences in movement demands exist between positions and teams even within the same university and during the same week of practice. For clinicians making decisions about re-entry into sport participation, our findings highlight the need to understand the unique demands to which the individual athlete is returning based on position, team, and practice type. We recommend that clinicians working with collegiate or other high-level athletes aim to approximate loading demands through video analysis of a practice, at the least using the simplified coding system we presented herein. For instance, a clinician viewing the data from this study may focus on ability to tolerate repetitive jumping for centers; running, cutting, and pivoting proficiency for forwards and guards; or the ability to tolerate intermittent bouts of high-intensity activity for members of the men's team. In contrast, a clinician returning a member of the women's team to practice may use the large amount of jogging in practices to lower the return-to-practice running criterion but be cautious about allowing full participation in jumping drills until that skill has been well-established off-court. Simplified loading categories can be used to monitor and appropriately remodel bone or soft tissue tolerance following a foot or ankle injury so that athletes do not relapse or re-injure themselves. Specifically, coupling known proportions with subjective reports of pain or objective assessment of swelling provide thresholds for practice capacity that can be used to precisely determine how much and what type of practice is allowed for an athlete returning from injury or during a period of increased susceptibility (e.g., bone stress reaction state).

## 5 Conclusion

Team and position influenced practice movement demands when characterized by either frequency or duration relative to total practice time for two NCAA Division 1 basketball teams. More differences were apparent between teams than among positions within teams, even though the practices were at similar points in the regular season and similar timeframes before a game. This finding emphasizes that team practice style and not just the player's positional demands should be considered when developing a rehabilitation program or RTS strategy. While the sample size precludes general statements about RTS demands for a given player, our study findings do provide an initial idea of possible position and team differences at the NCAA D1 level, particularly in light of the sparse data available on US collegiate basketball. Our findings emphasize that clinicians working with high-level athletes should consider team- and position-differences, quantify these whenever possible across a series of practices, and tailor clearance to return to practice accordingly.

## Data Availability

The raw data supporting the conclusions of this article will be made available by the authors, without undue reservation.
