# Supplementary material for: Physical demands of collegiate basketball practice: a preliminary report on novel methods and metrics
Source: Front Sports Act Living. 2024 Sep 23;6:1324650. doi: 10.3389/fspor.2024.1324650 (PMC11472002; doi:10.3389/fspor.2024.1324650)
Supplement: Supplementary file 1 [file Table1.docx]

Table 1. List of eight movement types coded from video and operational descriptors used to determine movement type.

| **Type** | **Operational descriptors** ^a^ |
| --- | --- |
| Stand/walk | - Activity**/steps** at no greater intensity than walking. No distinction between standing and walking. - No distinction made between standing and walking or between different intensities of walking. - Includes instances when a player is in a defensive stance: **either stationary or taking steps at an intensity no greater than walking.** - **Steps can be taken in any direction: forward, backward, or lateral but no shuffling occurs.** - **No flight phase (both feet never leave the ground).** - **Includes an offensive player performing a pick or a defensive player taking a charge. Both feet are planted in these cases.** - **Includes instances where a player is standing or stepping (but not shuffling) with no flight phase to hold/gain position underneath the net.** |
| Jog | - Movement (forward or backward) at intensity greater than walking but without urgency - **Includes skipping** - **Includes cross-over running, movement laterally with feet-crossing over each other, without urgency** - **Often moving in a general direction rather than a clearly defined target** - **Sharp change in direction can be achieved at this speed without deceleration or a lateral cutting movement** - **Upright torso angle** |
| Run | - Forwards/backwards movement at intensity greater than jog and a moderate degree of urgency but not approaching intense level of movement - **Includes cross-over running, movement laterally with feet-crossing over each other, with urgency** - **Often marked by moving toward a clearly defined target** - **Sharp change in direction cannot be achieved at this speed without deceleration or a lateral cutting movement** - **Torso can be either upright or slightly leaned forward** |
| Stride/sprint | - Forward movement at high intensity, effort and purpose at or close to maximum - **Increased knee drive near or past 90 degrees hip flexion** - **Often marked by dramatically increased forward trunk angle** |
| Low intensity shuffle | - Lateral or backward movement using shuffling action of feet - Without urgency, slow rate of foot movement and erect posture - **Often moving in a general direction rather than a clearly defined target** |
| Medium intensity Shuffle | - Shuffling at medium intensity with moderate level of urgency - Moderate rate of foot movement but not approaching an intense level of shuffling-type movement - **Often marked by moving with or toward a clearly defined target** - **Posture can be erect or slight squat position** - **Speed does not exceed the player’s jogging speed** |
| High intensity shuffle | - Shuffling at high intensity characterized by effort/urgency and rapid foot movement while usually in squat position - **Speed and urgency approach a range near, at, or past the player’s running speed. Player should be able to seamlessly transition into running (e.g., faster than the player’s jogging speed).** - **Includes instances where a player is driving into the other player while shuffling their feet** - Ground may not have been covered as the feet may have been shuffling rapidly on the spot or transferring weight from side to side |
| Jump | - Time from initiation of jump to completion of landing - **Both feet leave the ground with the player showing intent to increase their playing height and/or move vertically** |

Bold text indicates modifications and/or additions made to descriptors from original work of McInnes et al. (10) to improve classification and level of agreement among raters.

^a^We updated the McInnes definitions to provide specific descriptors that could allow raters to better discriminate varying intensities of movement patterns. For example, the differences between running and striding/sprinting were originally defined with descriptors such as “moderate degree of urgency” and “high intensity”. However, the subjective nature of these descriptors applied in varying contexts left situations open to varied interpretation between raters. Adding specific descriptors for trunk lean, hip flexion angle, and movement toward clearly defined targets vs. a general direction allowed raters to report their findings with a higher degree of reliability. Reference: McInnes SE, Carlson JS, Jones CJ, McKenna MJ. The physiological load imposed on basketball players. *Journal of Sports Sciences*. 1995;13:387-397.
